# Management of intertarsal septic arthritis in an ostrich (*Struthio camelus*)

**DOI:** 10.1002/vms3.643

**Published:** 2021-10-06

**Authors:** Melanie J. Peel, Rodrigo S. Garcés Torres, Marina Ivančić, Benjamín E. Alcántar Hernández

**Affiliations:** ^1^ The Veterinary Department, Wildlife Safari Winston Oregon USA; ^2^ Clinic for the Rehabilitation of Wildlife Sanibel Florida; ^3^ ZooRadOne Plainfield USA; ^4^ Present address: Department of Medicine and Epidemiology, School of Veterinary Medicine University of California Davis Davis CA 95616 USA

**Keywords:** avian, arthrotomy, *Corynebacterium*, ratite

## Abstract

A 7‐year‐old female ostrich (*Struthio camelus*) presented with lameness, left intertarsal joint swelling and a healing wound on the caudomedial aspect of the joint. Synovial culture revealed *Corynebacterium* species and radiographs were consistent with progressive septic arthritis. Multiple treatments were attempted including through‐and‐through joint lavage, intra‐articular antibiotics, caudomedial arthrotomy, and regional limb perfusion in conjunction with systemic antibiotics and analgesia. Euthanasia was ultimately performed due to prolonged recumbency and poor prognosis. This report describes novel therapies and a surgical approach utilized for treatment of intertarsal septic arthritis in an ostrich and exemplifies the poor prognosis described in other species presenting with non‐responsive septic arthritis of critical joints.

## Graphical abstract

A female ostrich (*Struthio camelus*) presented with left intertarsal septic arthritis that was culture positive for Corynebacterium. This report describes novel therapies and a surgical approach utilized for attempted treatment of intertarsal septic arthritis in an ostrich and exemplifies the poor prognosis described in other species presenting with non‐responsive septic arthritis of critical joints.

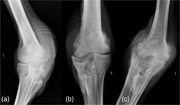

## 1 CASE PRESENTATION

A 7‐year‐old, 118 kg, female ostrich (*Struthio camelus*) with a body condition score of 5/9 presented with an acute grade 3 left limb lameness (Kestin et al., 1992). On distant visual assessment, the patient had intertarsal (tibiotarsal‐tarsometatarsal) joint swelling and a healing caudomedial wound just distal to the joint. The ostrich was housed in a free‐ranging exhibit with several species of African hoof stock.

**FIGURE 1:**
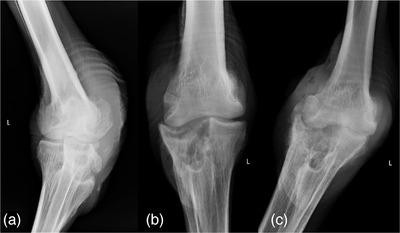
Radiographic progression of osteomyelitis following diagnosis of intertarsal septic arthritis in an ostrich (*Struthio camelus*). (a) Craniomedial‐caudolateral oblique projection of the left intertarsal joint, one day post‐clinical presentation. The “L” marker indicates the craniolateral surface. There is proximal and caudal soft tissue thickening, severe lysis of the caudal aspect of the proximal tarsometatarsus, and irregular bone fragments caudal to the joint. (b) Craniocaudal view of the left intertarsal joint 15 days post‐initial presentation. The “L” marker is on the lateral aspect of the limb. There is progressive severe soft tissue thickening and ongoing lysis of the proximal tarsometatarsus. Gas lucencies adjacent to the medial epicondyle of the tibiotarsus were attributed to iatrogenic introduction of air during arthrocentesis. (c) Craniocaudal view of the left intertarsal joint 48 days post‐initial presentation. The “L marker denotes the lateral surface. There is ongoing lysis, progressive soft tissue thickening, irregular periosteal proliferation along the proximal tarsometatarsus and development of joint space collapse.

Chemical immobilization was performed with tiletamine‐zolazepam (3 mg/kg IM, Dechra Veterinary Products, Overland Park, KS, USA) and medetomidine (0.2 mg/kg IM, ZooPharm, Laramie, WY, USA). Anaesthesia was maintained with isoflurane (VetOne, Boise, ID, USA), administered via an 18‐French endotracheal tube with supplemental oxygen. On examination, the left intertarsal joint was warm and exhibited significant palpable effusion. The wound on the caudomedial aspect exhibited no discharge or exposed tissues and had prominent callous formation. Using standard chlorhexidine and saline, the wound and joint were cleaned and aseptically prepared for arthrocentesis. An 18‐guage needle was inserted into an area of fluctuant swelling on the caudolateral aspect of the joint. Synovial fluid was grossly turbid, tan and exhibited decreased viscosity. A marked heterophilic rich exudate consisting of a mixed population of degenerate and toxic heterophils was seen utilizing a modified Romanowski stain (Rapid Differential Stain, VetOne, Meridian, ID, USA) on in‐house cytology with no pathogens noted. Radiographic evaluation revealed moderate circumferential soft tissue thickening of the intertarsal joint, with large osseous defects in the caudoproximal tarsometatarsus, multiple abnormal intra‐articular bone fragments, and proximal irregularity of the lateral condyle of the distal tibiotarsus (Figure 1a).

In attempts to eliminate inflammatory mediators and possible pathogens from the joint, a through‐and‐through joint lavage was performed (Mulon et al., 2016). Due to patient positioning and location of effusion, three 16‐gauge catheters were placed into areas of effusion on the craniomedial, craniolateral and caudomedial aspects of the intertarsal joint. The joint was kept in slight flexion throughout the procedure to allow for maximum joint space separation. Lavage was performed with saline (1000 ml Intra‐articular, 0.9% Sodium Chloride injection, Hospira, Lake Forest, IL, USA) followed by intra‐articular gentamicin (500 mg IA, VetOne, Boise, ID, USA). Catheter sites were allowed to heal by second intention. Bloodwork revealed no abnormalities, most notably, no elevations in white blood cell count. The patient was reversed with atipamezole (1 mg/kg IM, Zoetis Inc., Kalamazoo, MI, USA) and prescribed clindamycin (5 mg/kg PO q8h for 7 days, Aurobindo Pharma Inc., East Windsor, NJ, USA), enrofloxacin (2 mg/kg PO q12h for 10 days, Dechra Veterinary Products, Overland Park, KS, USA), phenylbutazone (10 mg/kg PO q12h for 3 days, VetOne, Boise, ID, USA) and tramadol (5 mg/kg PO q12h for 4 days, Sun Pharmaceutical Industries Inc., Cranbury, NJ, USA).

Lameness and effusion remained static over the course of two weeks following the initial presentation. On day 15 post‐presentation, radiographs and transarticular lavage were repeated under the same anaesthetic protocol mentioned previously, following collection of synovial fluid into a sterile, transfer media culturette for aerobic culture at a local laboratory (IDEXX, Memphis, TN, USA). Aerobic cultures were performed using both Macconkey and blood agars at 35°C (95°F). Radiographs revealed progressive severe soft tissue thickening, ongoing lysis of the proximal tarsometatarsus, and irregular periosteal proliferation (Figure 1b). Medications were continued as previously prescribed. Within 5 days, culture results were negative, however septic arthritis was still suspected.

**FIGURE 2:**
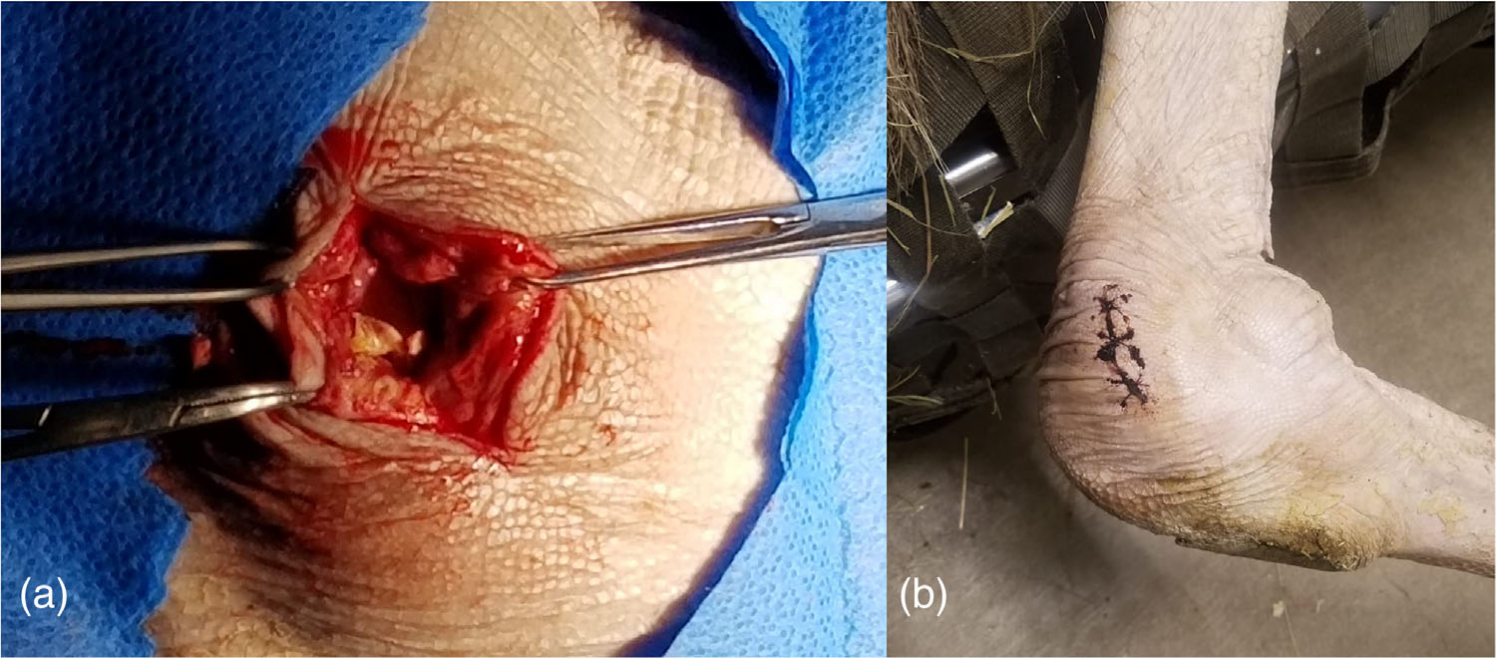
Caudomedial intertarsal arthrotomy approach in an ostrich (*Struthio camelus*) for management of septic arthritis. (a) Fibrinous and caseous material is seen within the intertarsal joint on initial caudomedial approach. Caudal is to the left of the photo. (b) Seven days post‐op healing of intertarsal joint arthrotomy.

On day 25 post‐presentation, the patient became acutely non‐ambulatory. More aggressive therapy was deemed necessary and an arthrotomy was performed using a novel surgical approach to the caudomedial aspect of the intertarsal joint. Standard arthrotomy principles were utilized for surgical planning from similar descriptions in cattle and an ostrich (Mulon et al., 2016; Tully et al., 1995). Anaesthesia was conducted using the technique mentioned previously and the patient was placed in left lateral recumbency, allowing for visualization of the medial aspect of the left pelvic limb. Aseptic preparation of the joint was performed in a standard fashion. A 5‐cm incision was made using a 10‐blade scalpel (VetOne, Meridian, ID, USA) on the caudomedial aspect of the joint exposing a copious amount of fibrinous and caseous material, which were collected in sterile transfer media and sent for repeat aerobic bacterial and fungal culture at Oregon Veterinary Diagnostic Laboratory (Corvallis, OR, USA). Fibrinous adhesions obstructed differentiation between facial planes and the joint capsule (Figure 2a). Once removed, palpable enthesophytes and osteophytes were noted along with hyperaemic periosteum. The joint space was lavaged with 2 L of lactated ringer's solution (1000 ml IA, Hospira, Lake Forest, IL, USA). Deep tissues were closed using 2‐0 PDS (Ethicon, Sommerville, NJ, USA) in a simple continuous pattern. Once deep tissues were closed, gentamicin (500 mg IA, VetOne, Boise, ID, USA) and polyglycan (6 ml IA, Bimeda Inc., Oakbrook Terrace, IL, USA) were deposited intra‐articularly, and skin was closed using 2‐0 PDS cruciates (Figure 2b). A standard regional limb perfusion with ceftiofur (2.5 mg/kg IV, Med‐Pharmex Inc, Pomona, CA, USA) and a tourniquet placed proximal to the intertarsal joint was also performed, utilizing the medial metatarsal vein as described in the avian model (Knafo et al., 2019; Ratliff & Zaffarano, 2016). The patient was recovered with atipamezole and treatment with tramadol, phenylbutazone, enrofloxacin and clindamycin were continued. Fungal cultures utilizing Sabouraud and blood agars at room temperature were negative. Aerobic bacterial cultures of synovial fluid and fibrinous material yielded profuse growth of *Corynebacterium* species. Both Macconkey and chocolate agars were placed at 35°C (95°F) to select for aerobic bacterial growth.

Following surgery, medication regimens were continued, and physical therapy was enacted. A sling was placed on the sternum and on the pelvis just caudal to the femur three times daily to aid in standing and passive range of motion for 5–10 min. Despite therapy, the ostrich remained non‐ambulatory and experienced a 30% weight decrease resulting in a 2/9 body condition score. On day 49 post‐presentation, radiography was repeated under anaesthesia which revealed progression of disease and development of joint space collapse (Figure 1c). Decubital ulcers were noted on the keel and posterior aspect of both intertarsal joints, and crepitus was present on the contralateral intertarsal joint due to development of arthritis. Due to progression of disease, poor prognosis and declining quality of life, euthanasia was elected. Gross necropsy and post‐mortem histopathology confirmed septic arthritis with osteomyelitis.

## 2 DISCUSSION

Septic arthritis is not an uncommon diagnosis in both exotic and domestic animals presenting with lameness (Amer et al., 2019; Baron et al., 2019; Harcourt‐Brown, 2002; Innes, 2016; Marcon et al., 2019; Mulon et al., 2016; Nairn, 1973). In birds, most diagnoses of this ailment are related to the extension of pododermatitis, traumatic events occurring near a joint or hematogenous spread of bacteria (Amer et al., 2019; Baron et al., 2019; Harcourt‐Brown, 2002; Knafo et al., 2019; Marcon et al., 2019; Nairn, 1973; Ratliff & Zaffarano, 2016; Youssef et al., 2019). There are few publications about the diagnosis and management of intertarsal septic arthritis in avian species, and none have been reported in an ostrich (Amer et al., 2019; Marcon et al., 2019; Nairn, 1973). Although surgical management of phalangeal septic arthritis in an ostrich has been described, the critical nature of the intertarsal joint (particularly in this large, flightless species) made this case a unique challenge (Burba et al., 1996). Minimal literature for this disease process in ratites makes both treatment and determination of prognosis difficult.

To the author's knowledge, this is the first published report of *Corynebacterium* septic arthritis in a ratite. *Corynebacterium* is a notoriously difficult to eliminate, gram‐positive, non‐spore‐forming bacillus (Boltin et al., 2009; Funke et al., 1997). This bacterium is typically an opportunistic, environmental pathogen that was likely introduced or migrated into the joint from the proximate wound (Funke et al., 1997).

Despite broad spectrum antibiotics and copious flushing, it is unlikely full clearance of this organism would have been achieved. In mammalian species, necessary therapies for septic arthritis due to environmental pathogens, such as *Corynebacterium* and *Arcanobacterium*, often require a combination of transarticular lavage, systemic and intra‐articular antibiotics, arthrotomies, and intra‐articular drains (Desrochers, 2004). Due to avian physiology and the nature of the species, a drain was not a viable option (Smith et al., 1998). Avian abscessation is not conducive to active drainage, and formation of caseous exudate likely resulted in an environment which harboured the bacterium (Smith et al., 1998).

This report also presents novel therapies not previously described in ratites. A regional limb perfusion (RLP) was performed to aid in the treatment of septic arthritis by increasing drug concentrations in tissues not well vascularized (Knafo et al., 2019). In recent years, this technique has been briefly described and utilized in the avian model, allowing for extrapolations to be made for ratite medicine (Knafo et al., 2019; Ratliff & Zaffarano, 2016). The technique for RLP is relatively simple and should be considered for aggressive treatment of septic arthritis and other inflammatory conditions of the distal extremity in ratites.

An arthrotomy was also performed utilizing a novel caudomedial approach based on lesion location. A similar technique has also been described for treatment of a distal tibiotarsal bone cyst in an ostrich utilizing a dorsomedial approach (Tully et al., 1995). In ostriches, nearly all vasculature, ligaments, tendons and nerves are located on the posterior, anterior, medial or lateral surfaces of the distal extremity, allowing for oblique approaches to the joint (Hutchinson et al., 2015; Smith et al., 1998). The distal articular surface of the tibiotarsal bone is convex, with medial and lateral condyles of the tibiotarsus articulating with the concave cotyles of the proximal tarsometatarsus. This anatomy can lead to decreased visualization of all articular surfaces (Hutchinson et al., 2015; Schaller et al., 2009). Caution should be taken if attempting to locate a lesion in the central portion of the articular surface (i.e., the intercondyloid fossa).

Most importantly, determining integrity of the intertarsal joint is critical for treatment success. There are complex ligamentous and osseous anatomical structures needed for proper biomechanics of ostrich ambulation, including the engage‐disengage system (Schaller et al., 2009). For appropriate range of motion, the ostrich intertarsal joint require at least 168° of extension and 80° of flexion, achieved during daily physical therapy for this patient (Schaller et al., 2009). The menisci, tendons and ligamentous apparatus are necessary for swing phase mechanics and passive locomotion that allow for decreased energy expenditure and appropriate movement (Schaller et al., 2009). Pathology involving this apparatus can lead to inefficient or complete loss of ambulation, as seen in this case. More specifically, osteomyelitis affecting the articular surfaces in conjunction with severe fibrinous adhesions disrupting ligamentous and joint capsule integrity contributed to causes of prolonged recumbency and decubital sequelae. The complex nature of the intertarsal joint of ostriches and other ratites may be a limiting factor when managing non‐responsive septic arthritis. Improved clearance of pathogens within the joint may be possible with more aggressive therapies, such as an arthrotomy and RLP, at time of presentation. Caution is advised if significant osteomyelitis involving the articular surfaces has already occurred, as return to full function may not be possible.

## 3 CONCLUSION

The novel therapies utilized in this report are areas of interest for further research to determine efficacy of regional limb perfusions and success of arthrotomies in ratites. Additional work is also needed in determining prognosis of infections involving *Corynebacterium* and cases of non‐responsive septic arthritis of the intertarsal joint.

## CONFLICT OF INTEREST

The authors report no interest in production of this manuscript.

## ETHICS STATEMENT

The authors confirm that the ethical policies of the journal, as noted on the author guidelines page, have been adhered to. No ethical approval was required as this was a case report.

## AUTHOR CONTRIBUTIONS

Melanie J. Peel: Conceptualization; Data curation; Investigation; Methodology; Project administration; Writing‐original draft; Writing‐review & editing. Rodrigo S. Garcés Torres: Conceptualization; Data curation; Investigation; Methodology; Project administration; Writing‐review & editing. Marina Ivančić: Formal analysis; Writing‐original draft; Writing‐review & editing. Benjamín E. Alcántar Hernández: Conceptualization; Investigation; Methodology; Project administration; Writing‐original draft; Writing‐review & editing

### PEER REVIEW

The peer review history for this article is available at https://publons.com/publon/10.1002/vms3.643

## Data Availability

All relevant data are within the paper and supporting information files.
